# Adapting E-cigarette prevention programming to reach the latinx community

**DOI:** 10.1007/s10552-023-01796-7

**Published:** 2023-10-09

**Authors:** Alison K. Herrmann, Sylvia Lopez Ferullo, Miriam Hernandez, Verónica Arciga Barriga, Bernadett Leggis, Julissa Ruiz, Beth A. Glenn, Roshan Bastani

**Affiliations:** 1 UCLA Center for Cancer Prevention and Control Research, UCLA Kaiser Permanente Center for Health Equity, Department of Health Policy and Management, Fielding School of Public Health and Jonsson Comprehensive Cancer Center, University of California Los Angeles, 650 Charles E Young Dr. S, Los Angeles, CA 90095-6900 USA; 2 UCLA Center for Cancer Prevention and Control Research, UCLA Kaiser Permanente Center for Health Equity, Fielding School of Public Health and Jonsson Comprehensive Cancer Center, University of California Los Angeles, 650 Charles E Young Dr. S, Los Angeles, CA 90095-6900 USA; 3 Visión y Compromiso, 1000 Alameda Street, Los Angeles, CA 90012 USA

**Keywords:** Vaping/e-cigarettes, Promotor/as, Latinx, Parent education, Community-based prevention program

## Abstract

**Purpose:**

E-cigarettes are the most commonly used tobacco product among youth in the United States. Yet evidence-based prevention programming is limited due to the rapid onset of this threat. Community-based efforts to address vaping largely target youth in school settings. Although parents can play an important role in youth tobacco control efforts, messages about the dangers of vaping, use among adolescents, and strategies for intervening have not reached many Spanish-speaking parents in low-income Latinx communities. Our community-academic team developed e-cigarette prevention programming for use by promotor/as de salud to address this unmet need.

**Methods:**

During the 1-year project, the team worked closely with a Project Advisory Committee to: review existing evidence-informed materials; conduct focus groups with parents, youth and promotor/as to guide program development; develop a curriculum to prepare promotor/as to educate low-literacy, Spanish-speaking parents about vaping; craft Spanish language resources for promotor/as to use in community education sessions; train 61 promotor/as to deliver the program; and support program delivery to 657 community members.

**Results:**

Focus groups with promotor/as and community members, key-informant interviews, and brief surveys informed program development and assessment. Community member feedback was essential to development of appropriate materials. Promotor/as demonstrated significant pre- to post- training increases in e-cigarette knowledge and confidence in delivering vaping prevention education. Community members demonstrated a mastery of basic e-cigarette concepts and expressed intention to discuss vaping with their children.

**Conclusions:**

Promotor/a-led programming for parents represents a promising approach to vaping prevention and control in the Latinx community.

**Supplementary Information:**

The online version contains supplementary material available at 10.1007/s10552-023-01796-7.

## Background

E-cigarette use/vaping has emerged as a serious national health concern, threatening to roll back decades of prior gains in tobacco prevention and control [1]. Since 2014, e-cigarettes have been the most commonly used tobacco product among youth in the United States [2, 3]. Evidence-based vaping prevention programs are being developed [4, 5], largely in the form of school-based interventions [6–8]. These efforts represent a promising step in vaping prevention and control. However, many parents of youth who are at risk of using these products are not well informed about the use and dangers of these products among children [9]. This gap in knowledge is likely most pronounced among parents with limited levels of education and English proficiency. As a result, large groups of parents remain unprepared to identify or address the issue with their children, which may exacerbate existing health disparities.

Community Outreach and Engagement (COE) teams at NCI-designated Cancer Centers are charged with disseminating evidence-based information and programs to the diverse communities within their catchement areas and to responding community-identified needs and priorities [10]. These recipricol relationships between the university and community encompass a range of projects and programs. Early efforts to be responsive to expressed community needs often inform, and are preliminary to, more rigorous research endeavors. The 1-year project described here represents this type of preliminary step. We conducted this work in response to numerous requests for e-cigarette programming. received from members and leaders in the communities served by the UCLA Jonsson Comprehensive Cancer Center (JCCC). These requests highlighted a need for programming that is culturally and linguistically appropriate for the Spanish-speaking Latinx community, that would increase parental knowledge and empower parents to address this important health issue with their children.

Interveening with parents is an approach recommended by the Community Preventive Service Task Force based on evidence from a systematic review demonstrating improvements in a range of adolescent risk and protective health behaviors, including tobacco use [11]. Studies included in the review shared the following components: education, discussion, and opportunities to practice skills, which we incorporated in this project. More recent research has also demonstrated the effectiveness of parent-focused interventions with regard to adolescent risk behaviors including substance use [12] as well as a significant relationship between childrens’ perceptions of parental knowledge and their risk of vaping such that youth who believed their parents are more knowledgable were less likely to vape at both 6 and 12-month follow-up.[13]. These interventions not only provide parents with information, but also assist them in navigating challenges associated with communicating with their children about sensistive topics.

*Promotor/a* support agencies with whom the JCCC routinely collaborates expressed a desire for vaping education programming so that promotor/as could share accurate information with the Spanish-speaking Latinx community members they serve. These individuals and organizations have enduring relationships in local communities and are trained and trusted messengers of health information and programming. There is strong evidence for the effectiveness of promotor/a-led programming targeting vulnerable communities across a wide range of cancer prevention and control outcomes [14–22] and prior research has documented the effectiveness of parent-focused interventions that employ trained individuals, such as health coaches, in improving adolescent health behaviors [11]. COE efforts at the JCCC include multiple community programs delivered in close collaboration with promotor/as and agencies that support them. As the academic partner in this work, our role includes training promotor/as to ensure that they are knowledgable about the health topic to be addressed through programming with community members. This approach is central to our ability to effectively reach the population of Los Angeles County, the JCCC’s catchment area.

Los Angeles is the most populous and diverse county in the United States. Latinos comprise the largest ethnic group in the county (49%) and 60% of county residents under the age of 18 years are Latinx. Nearly half of Latinx adults in the county are foreign-born (49%), 32% have less than a high school degree, and 14% live below the poverty line [23]. Nearly one-third of Los Angeles County high school students had tried e-cigarettes and one in ten were current users, based on the most recent available data from 2017 [24]. National data indicate that vaping among youth may have peaked in 2019, but the practice remains common. Greater than 20% of youth continued to report vaping in subsequent years, with a slight increase observed from 2021 to 2022 (22.1 to 23% across grades 8, 10 and 12 combined) [25]. Among middle and high school students in Los Angeles County who have never tried other tobacco products, those who identify as Latinx are more likely to use e-cigarettes than non-Latinx whites [26]. Additionally, data from the most recent National Youth Tobacco Survey suggest that Latinx youth may initiate e-cigarette use earlier than non-Latinx white youth [27]. The health concerns associated with these data, coupled with the JCCC COE team’s commitment to addressing community-identified needs, led to a community-academic partnership aimed to develop promotor/a-led vaping education for Spanish-speaking, Latinx parents of adolescents, and to prepare promotor/as to deliver this programming. Given the size of the county, we decided to focus our initial efforts on the Antelope and San Fernando Valley regions of the county, home to large numbers of lower-income monolingual Spanish speakers and where tobacco use and vaping are more prevalent compared to other regions of the county [24]. The process we used in conceptualizing and planning this project were similar to those of Intervention Mapping, which takes a step-by-step approach from identification of a health issue and focus population, development of program objectives, selection of methods and strategies, intervention development, implementation planning and evaluation [28, 29]. We aimed to: (1) idenfiy and adapt evidence-informed e-cigarette education and prevention programming for use by promotor/as with Spanish-speaking parents of Latinx youth; (2) develop a training to prepare promotor/as to address e-cigarettes with parents and provide this training to promotor/as in the Antelope and San Ferndando Valley regions of Los Angeles County; (3) support promotor/as in delivering e-cigarette programming with parents; (4) evaluate the program with promotor/as and community members.

## Methods

To address the identified need for tobacco and vaping education among Spanish-speaking parents of Latinx youth, the JCCC’s COE team partnered with Visión y Compromiso (VyC; visionycompromiso.org), a community-based agency with a 20-year history of providing training and skill building activities to prepare promotor/as to address a wide range of health issues in vulnerable communities. Building on a strong foundation of prior and current collaborative health promotion efforts, UCLA and VyC sought to develop a comprehensive promotor/a training curriculum and resources to support promotor/a-led e-cigarette education and prevention activities for Spanish-speaking, Latinx parents of adolescents. To ensure that the program was culturally and linguistically appropriate, we developed all materials in Spanish with subsequent translation to English, as needed. We sought to evaluate the outcomes of the training program on promotor/as’ vaping knowledge, to assess the feasibility of delivering promotor/a-led vaping programming for parents, and to assess the acceptability of this programming and its effect on knowledge among community members. Given the pronounced need identified in the Antelope and San Fernando Valley regions in the northern portion of Los Angeles County, we elected to focus our efforts within this geographic area, with the intention of disseminating our findings to other regions in the county, state and beyond. This project was conducted from April 2020 to March 2021; human subjects approval was obtained from the University of California, Los Angeles (UCLA) Institutional Review Board (IRB; #20-001734). Sixty-one promotor/as participated in the project. Education sessions were attended by 657 community members, 353 of whom (53.7%) participated in evaluation activities. Our work was supported by a supplemental funding award to the JCCC’s Cancer Center Support Grant provided by the National Cancer Institute (NCI). This funding opportunity aimed to understand how COE programs at NCI-designated Cancer Centers can work with community partners to identify, adapt, and implement existing evidence-based interventions, to meet the needs of the communities that cancer centers serve. We describe steps taken in our community-partnered program development, delivery and evaluation process in chronological order below.

### Project team and roles

All aspects of this work were undertaken by a project team comprised VyC staff, UCLA staff, and a nine-member Project Advisory Committee (PAC) convened at the outset of the project by VyC and UCLA. The PAC included promotor/as, leaders of promotor/a agencies, tobacco researchers, as well as tobacco control leaders from the Antelope and San Fernando Valley regions of Los Angeles County. Meeting quarterly over the project period, via Zoom, with additional email and phone communication as needed, the PAC provided in-depth guidance throughout all phases of the project. The VyC group included a regional promotor/a network manager, a promotor/a training lead who formerly practiced as a physician in Mexico, and a project coordinator. This group played a key role in recruitment of promotor/as and community members for participation in all program activities, coordinated focus groups and promotor/a trainings and actively supported the promotor/as throughout program delivery. The UCLA group included health services researchers as well as COE staff. This group led the process of identifying existing resources for review and adaptation, developed draft program materials, and used an iterative process to adapt materials in accordance with guidance received. The UCLA group also led the development and implementation of program evaluation activities, including pre- and post-program focus groups, brief surveys to assess impact among promotor/as and program participants, and post-program key-informant interviews. Promotor/a trainings were delivered by UCLA and VyC. The full project team worked closely in the assessment and adaptation of program materials, and in the interpretation of evaluation data as well as the dissemination of project results. Below we provide a chronology of the steps we took in this project.

### Scan of existing resources

The first step for the project team was to conduct a scan of available evidence-informed e-cigarette and tobacco prevention materials/programs. We conducted literature reviews via PubMed and internet searches of the grey literature. Our PAC provided additional guidance regarding publicly available programs. We identified evidence-informed resources for review that were developed by the American Lung Association, Scholastic and the Food and Drug Administration, the Partnership to End Addiction, Parents Against Vaping (PAVE), Flavors Hook Kids, Stanford University (Stanford Tobacco Prevention Toolkit) and other agencies. The community and academic project team reviewed these resources, seeking to identify any program components that were appropriate for adaptation and use with our intended audience. We identified some limited appropriate content elements, which helped to guide the selection of our program modules, but were unable to locate any existing comprehensive curricula suitable for use in training promotor/as. We did not find any complete community-facing programs that our community advisors considered appropriate for use among Spanish-speaking community members with limited levels of education.

### Initial draft of program materials

Given this lack of existing materials appropriate for use with our target audience, we elected to develop new resources specific to our program. With regard to promotor/a training, we drafted sample content for a PowerPoint presentation and a print training manual including vaping and tobacco use statistics, health effects of product use, and the role of marketing in promoting use. We also drafted sample content for a flip chart to be used by promotor/as in small group educational sessions with an emphasis on visual images to be viewed by community members that clearly illustrate the concepts to be communicated. All draft materials were developed in Spanish by bilingual, native Spanish speakers in the UCLA group, with guidance from the PAC and VyC. Drafts included the latest evidence in topic areas shared across existing programs (e.g., dangers of tobacco use, chemicals included in e-cigarette liquids), using simplified language and images, colors and fonts that were identified by the PAC and VyC to be most appropriate for our promotore and community audiences.

### Focus groups to guide materials development (n=6 groups)

Feedback on draft program materials was obtained through a series of six focus groups. Two groups were conducted with Latinx middle and high school students, two with Spanish-speaking parents of adolescent children and two with promotor/as. VyC recruited students and parents from community settings in our geographic focus regions and promotor/as through their own network and affiliates. Groups were facilitated by bilingual research team members, the content recorded, transcribed and translated to English as needed. Groups with students were conducted in English and all other groups were conducted in Spanish. Due to the onset of the COVID-19 pandemic, all groups were held via Zoom. A total of 13 youth (7 high school; 6 middle school), 12 parents (5 from the Antelope Valley; 7 from the San Fernando Valley) and 13 promotor/as (7 from the Antelope Valley; 6 from the San Fernando Valley) participated in the focus groups. Topics addressed in focus groups included: knowledge and attitudes about vaping, vaping terminology, parent/caregiver and child communication, and feedback on draft program materials (e.g., language use, colors, images). Groups with promotor/as also focused on feasibility of using the materials we had drafted in the community. Groups with students were conducted first, followed by the parent groups and lastly the promotore groups. To gain maximum benefit from the groups, we continually modified our resources based on what we learned as each group took place such that information about the terminology students use to talk about vaping was incorporated into draft materials reviewed by parents and common misconceptions among parents were incorporated into materials reviewed by promotor/as, etc. Each transcript was prepared and analyzed by UCLA immediately following a group session, using standard word processing software. Key themes were identified and reviewed with VyC and the PAC, with input from all project team members, and were also used to revise the draft materials considered by subsequent groups. This iterative approach allowed us to incorporate feedback obtained from each group and continually update and improve our program content. Through this process, the project team developed a final PowerPoint slide deck and print training manual for use in the promotor/a training sessions, and a flip chart for promotor/as to use in their community education sessions. We consider these materials to be a central outcome of this project and as such they are described in the Results section.

### Promotor/as training

We scheduled four 2-day (6–8 h per day) promotor/a training sessions. VyC promoted the opportunity for this training within their existing promotor/as network. Sixty-one promotor/as completed both days of the training (51 from the San Fernando Valley and 10 from the Antelope Valley; 56 women and 5 men); six of the 67 promotor/as who participated in the first day of the training were unable to attend the second day due to scheduling issues or illness. Promotor/as who completed the training did so as a cohort; there was no cross-over between the four groups. Three of the 2-day training sessions were conducted in-person with the remaining session conducted over Zoom due to safety concerns associated with the COVID-19 pandemic. Pre-post-training surveys were used to assess promotor/as’ knowledge about vaping (23, true/false items) and included a single item to assess promotor/as’ confidence in leading a vaping education session in the community [on a scale of 1 (not confident) to 4 (very confident)]. Surveys were administered at the start of the first day of training (pre) and immediately following completion of the second day (post). We developed the training to ensure that promotor/as had the necessary knowledge and skills to deliver the program as a part of their usual health education activities in the community. This approach is in keeping with the type of trainings that VyC typically provides for the promotor/as, both volunteer and paid, that the agency serves. VyC identified and hired five of the trained promotor/as (4 from the San Fernando Valley; 1 from the Antelope Valley) to deliver the program as part of this project.

### Program delivery

The five project promotor/as led 64 community sessions (charlas) over a three-month period. A total of 657 community members (400 San Fernando Valley; 257 Antelope Valley), all Spanish-speaking parents/caregivers of adolescents/teens, attended these sessions. Promotor/as recruited participants in community settings, leveraging their personal networks. Sessions were delivered over a three-month period with the goal of including as many community members as possible during that time frame. Fifty-three of the sessions were held virtually, via Zoom. Approximately half (*n* = 33) of the sessions were conducted with parents recruited through school sites. The remaining participants were recruited at other community venues. While recognizing the importance of evaluation, we were especially cognizant of the need to minimize respondent burden. We therefore decided to conduct a brief (5-item) on-line post-session survey to assess knowledge regarding e-cigarette contents, health effects of vaping, industry marketing tactics, communication approaches, and intention to discuss vaping with their child/ren. We developed items using simple, lay language consistent with that included in the program and binary (yes/no) response options. We selected the online survey format in order to maintain a uniform process for all participants (virtual and in-person). All program participants were eligible to complete the survey. Interviews were conducted with a sub-set of volunteer participants (*n* = 50) immediately following the session in order to solicit more in-depth feedback about the aspects of the program that they found most beneficial and potential areas for program improvement. The interviews were conducted in-person or via Zoom in keeping with the session format. Up to two participants in each session were included in the interview process.

### Post-program focus groups (n = 3 groups)

Following the 3-month program delivery period, a final series of focus groups was conducted via Zoom with promotor/as; those who completed the 2-day training and had delivered at least one community education session were eligible to participate. Of the three groups, one was specific to the promotor/as who were hired to deliver the program for this project. These groups were conducted to gain an understanding of promotor/as’ experiences recruiting participants and leading educational sessions as well as to learn how acceptable the promotor/as perceived the program to be among community members.

### Data analysis

Data from focus groups with community members and promotor/as were analyzed by the study team with an emphasis on identifying themes that could guide program development and refinement, using an directed approach [30] consistent with rapid qualitiative analysis [31–35], in keeping with iterative nature and quick turn-around time required in this work. Analyses of survey data gathered from community members and promotor/as were primarily descriptive in nature, with bivariate analyses (one sample *t* test) conducted to examine the significance of changes in promotor/as’ knowledge from pre- to post-training. Qualitative analyses were conducted using standard word processing software; quantitative analyses were conducted in SAS, version 9.4 [36].

## Results

### Findings from focus groups to guide program development

Information obtained through the pre-program focus groups with youth, parents and promotor/as was key to shaping the content of all program materials, including terminology, images, explanation of complex concepts, and specific messages that were important to convey. Feedback from youth was especially helpful in determining the most commonly used vaping products in the community and terminology used to reference these products. Youth also shared that they learn about vaping through social media and their friends and that they obtain products by purchasing online, at local shops, or from their friends. They reported that ongoing use was related to stressors in the family/home environment and indicated that vapes/vaping is very easy to hide and many parents are “clueless” that their children are vaping. When asked about strategies that parents could use to discuss vaping with their children, youth indicated that it would be especially important for parents to listen without judgement. Groups conducted with parents and promotor/as underscored a lack of awareness and knowledge surrounding vaping among young people in the community. Sessions with parents revealed hesitation to discussing, in a group setting, the possibility of their child vaping, given stigma in the community associated with having a child who uses tobacco products as a potential barrier to open discussion among group participants. All groups emphasized the importance of providing parents in the community with guidance as to how they could approach this issue with their children.

### Program resources developed through this project

Building upon draft materials, what we learned through the pre-program focus groups and guidance from our PAC, we developed a 6-module, interactive promotor/a training curriculum (see Table 1). Modules 1–4 were included in the first day of the training. Modules 5 and 6 were included on the second day. Modules were designed to be delivered in approximately one hour, with the specific time devoted to each module ranging from 45 to 75 min. Homework was assigned at the end of Day 1 and reviewed at the start of Day 2. This format was consistent across the in-person and Zoom training sessions. We crafted the program flip chart specifically to aide promotor/as in conveying the key concepts presented in modules 1–5 when leading sessions with community members. Community facing pages of the flip chart were largely visual. Corresponding written notes faced the promotor/as to guide their presentation. We maintained major topic areas and important facts (e.g., youth vaping statistics) included in other programs in our materials. We incorporated bright colors throughout all materials and took our own photographs with local community members for several of the images given feedback that colors and images included in other materials may not resonate with the community members we sought to engage. Based on parents’ and promotor/as’ feedback, the flip charts in particular were developed to be highly visual, with few words and only simple language included. Terminology used to reference vaping (vapear, fumando) was determined based upon feedback obtained from youth. See supplemental materials for English translations of sample content from the training manual and flip chart.

**Table 1:** Summary of Content Included in Promotor/as Training Curriculum

Training modules	Main themes	Key topics addressed
Module 1: E-cigarettes and vaping	Vaping basics	Types of devices
		How vapes work/function
		Ingredients in vapes (nicotine, flavors, chemicals)
		Language youth use to talk about vaping
Module 2: Vaping among youth	Youth epidemic	Youth vaping statistics
	Appeal to youth	Why youth vape
		How youth access vapes
Module 3: Hooking a new generation	E-cigarette marketing	Advertising directed toward youth
		Social media marketing
Module 4: Health concerns	Health impacts	Nicotine
		Addiction
		Aerosol and toxic chemicals
		Health impacts on brain, heart and lungs
Module 5: What parents can do	Tips/Resources for parents/families	Signs of vaping
		Parent–child communication
Module 6: Promoting vaping awareness	Practice for delivering community sessions	Main points for community sessions (charlas)
		Flipchart review and practice
		Vaping education and cessation resources

### Findings from pre-post-training survey with promotor/as

All 61 promotor/as who attended both days of the training completed pre- and post- training surveys (see Table 2). Promotor/as’ demonstrated significant pre- to post- training increases in knowledge; *t*(60) = 6.9691, *p* < 0.001. On average, overall survey scores calculated based on the 23 knowledge items increased nearly 11% points (*M* = 10.6, SD = 1.75) from pre- to post-training. The average post-program score was 86%, corresponding to 20 correct responses. Prior to the training, the only item that was correctly answered by all promotor/as was that nicotine is highly addictive. Following the training, all promotor/as were able to correctly answer seven of the items. The largest pre-to post-program improvements were observed in items assessing where teens purchase e-cigarettes (Teens typically obtain electronic cigarettes at supermarkets; 32% correctly responded false pre-training vs. 72% post-training) and current understanding of long-term health risks (not much is known yet about vaping’s long-term health risks; 26% correctly responded true pre-training vs. 82% post-training). The final survey item assessed promotor/as’ readiness to deliver e-cigarette programming. Following completion of the training, 82% of promotor/as (*n* = 50) indicated that they were very confident in their ability to lead a vaping education session with community members using the flip chart developed by the study team, an increase from the 50% who expressed such confidence prior to the training.

**Table 2:** Proportion of correct survey responses provided by promotor/as before and after completion of 2-day vaping education training promotor/as (*n* = 61)

Survey item (yes/no)	% of Promotor/as responding correctly pre-training	% of Promotor/as responding correctly post-training
Electronic cigarettes are used to inhale nicotine, cannabis or other substances into the lungs	91	100
All electronic cigarettes are the same shape and color	93	100
Electronic cigarettes produce a harmless water vapor	87	100
Electronic cigarettes are easy to spot in adolescents’ backpacks	75	88
Traditional cigarettes are the most commonly used tobacco product among teens in the United States	51	74
Electronic cigarettes are the most commonly used tobacco product among teens in the United States	81	100
Nicotine and cannabis vaping has increased among teens in the U.S. in recent years	94	93
The most popular flavors for electronic cigarettes include fruit, mint, menthol, and candy or dessert	96	98
The main reason Latino adolescents use electronic cigarettes is curiosity	75	94
Teens typically obtain electronic cigarettes at supermarkets	32	72
Flavors in electronic cigarettes hide the taste of nicotine	85	100
The vaping industry’s advertising is targeting young people so that they may become consumers for many years	94	98
Very few electronic cigarettes contain nicotine	86	95
Nicotine is highly addictive	100	100
Nicotine and marijuana affect the development of young people’s brains	97	100
The aerosol in electronic cigarettes can cause irritation and inflammation of the airways	86	100
Not much is known yet about vaping’s long-term health risks	36	82
Electronic cigarettes contain many of the same toxic chemicals that traditional cigarettes do	82	88
Signs of vaping (the use of electronic cigarettes) can include fruity or sweet smells, unfamiliar items or products, and changes in behavior and mood	89	98
Parents and primary caregivers should speak firmly to their children about vaping	15	59
Before starting a conversation, parents and primary caregivers should become informed about vaping and take the time to practice	95	97
Parents and primary caregivers should only have one conversation about vaping with their child	83	95
If your child is using electronic cigarettes, it is important to understand why they are vaping and help them quit	98	98

### Findings from post-session survey with community members

The five-item post-program surveys were completed by 353 community members following participation in the group sessions. Nearly three-quarters (72%) of these individuals answered all four true/false knowledge questions correctly, with 17% correctly answering three of the four questions and another 9% correctly responding to two of the items. Table 3 lists the four knowledge items and the proportion of correct responses received. The fifth item asked when participants intended to have a conversation with their child/ren about vaping; 82% responded “within the next week”, 3% “within the next month”, whereas 13% indicated that they did not know and 1% (*n* = 4) said they did not plan to talk with their child/ren about vaping.

**Table 3:** Proportion of correct post-session survey responses provided by latinx community members who participated in promotore-led vaping education sessions

Survey item (true/false)	% of community members that responded correctly (*n* = 353)
E-Cigarettes produce harmless water vapor	81
The vaping industry has targeted teens though youthful advertisements and social media	94
Nicotine can cause long-term harm to the brains of young people	98
Parents only need to have a conversation about vaping with their children one time	86

### Major themes idenfitied from key informant interviews

Post-program key-informant interviews (*n* = 50) revealed that community members found details about e-cigarette devices including what they look like and how they work, strategies for parent–child communication, and information about the health risks associated with e-cigarette use to be the most helpful components of the program. Selected quotes reflecting participants’ experience include:

“I will talk to my child in the next week. I found all this information important and really impactful so, I think my husband should attend the next charla being held at the school so we can talk to our son together.” — Parent/Caregiver, post-session interview

“The presentation showed us photos of devices that are most commonly used and how they are used. When I saw them, I realized I had seen them [devices] before and they were being used by people I know. I took a screenshot and called my friend and I asked if she knew what it was and how dangerous it was to use. My friend said she thought it was harmless. I was just so surprised.” — Parent/Caregiver, post-session interview

“I think this presentation is so important for parents. I feel like when I am just getting to a topic... my children have already been there and came back. There's so much I need to learn and will continue to learn to let [my children] know.” — Parent/Caregiver, post-session interview

### Findings from post-program focus groups with Promotor/as (n = 3 groups)

Eleven promotor/as participated in post-program focus groups aimed to better understand their experiences with program delivery. 4 of the 5 promotor/as hired to deliver the program were available and took part in one group; these promotor/as had conducted between 7 and 23 community sessions. Seven promotor/as who compelted the two-day training and delivered at least one session also took part in this activity (four in one group; three in the other); these promotor/as had conducted between 1 and 5 community sessions. In each of the focus group discussions, promotor/as noted that parents in their education sessions were receptive to the material presented. All promotor/as indicated that they felt confident in delivering the material. However, several promotor/as shared that they were less comfortable delivering sessions virtually than in-person and that the need to conduct sessions via Zoom presented an added challenge for both recruitment and program delivery. All promotor/as indicated that the materials were easy to use and well received by the community members in the sessions they had conducted. They reported that many parents expressed shock in learning what vaping devices look like and how easy they are to hide, and that this was among the most compelling information presented in the educational sessions. Promotor/as noted that parents expressed great interest in opportunities to see actual vaping devices, versus only the photos of devices that were shown during the sessions. In each focus group, promotor/as also noted that the time dedicated to strategies that parents can use to communicate with their children about vaping was especially valuable. The promotor/as explained that communication presents a challenge for many parents in this community, with the tendency to focus on punishment versus prevention, a theme that came up in many of the group sessions. Promotor/as shared that parents were appreciative of the opportunity to discuss communication challenges in the groups and to explore ways to talk with their children in an open and non-confrontational manner.

## Discussion

NCI-designated cancer centers have a responsibility to serve communities in their catchment areas with evidence-based cancer prevention and control programs. Given that community health needs and threats are continually and rapidly evolving, there is sometimes a need to respond before comprehensive programs have been developed and rigorously evaluated. This becomes even more pressing for cancer center’s COE teams when community partners present with requests for assistance in addressing what they perceive to be among the most serious health issues facing the populations with whom they work. In such instances, cancer centers must seek to identify and adapt evidence-informed resources or programs to meet the needs of their catchment area communities, develop new materials where called for, and utilize evidence-based approaches to deliver this programming. Our work to address vaping in the Latinx community of Los Angeles County reflects such an approach. We utilized an evidence-based cancer prevention and control strategy, promotor/a-delivered programming, to address the need for vaping education among Spanish-speaking parents in the community, drawing from evidence-informed resources for use in this work.

In response to multiple requests for vaping education programming for Spanish-speaking community members, we worked closely with our community partners and advisors to engage promotor/as in the delivery of such programming. Community engagement, feedback and use of an iterative approach to incorporate this guidance was integrated throughout each stage of our program development process. Our work was conducted in close keeping with an Intervention Mapping approach [28, 29]. In future efforts, we believe it will may be helpful to more directly follow a framework such as this, to guide both our formative work and later research. Moreover, training community partners to use formal frameworks as a guide for program development and delivery falls well within our COE team’s focus on building partners’ capacity to address community health issues.

In keeping with the goal of ensuring that community members were able to fully engage in each phase of the work, we conducted all aspects of the project in Spanish with translation to English as appropriate. Despite recognized challenges related to the appropriateness of health education materials and programs for audiences with limited English proficiency that are first developed in English and then translated to community members’ preferred language [37–39], this approach remains common. We are hopeful that other COE teams across the nation will consider crafting programming as we did, in community members’ preferred lanaguge, to help ensure programs reflect the specific cultures and languages of the diverse populations they aim to serve.

Our work also highlighted the importance of nurturing and sustaining close relationships with community partners. COE teams engage with local communities in activities that span the continuum from outreach to shared leadership [40]. Authentic community engagement and recipricol parterships are well recognized for their value in serving communities and increasing health equity [41–44], and the lengthy time commitment needed to develop these types of relationships has been documented in the literuature [43, 44]. Engaging community members and promotores as we did in this project would not have been possible, particularly within the 1-year time frame allotted and in the face of the unique challenges posed by the COVID-19 pandemic, without Visión y Compromiso’s existing relationships in the community and their already well-established partnership with the UCLA JCCC.

The COVID-19 pandemic had significant implications for our work. It was necessary to modify our original plans for in-person promotor/a training and program delivery in the community such that most of these activities took place via Zoom. Similarly, post-session surveys completed by community members needed to be administered online, which many community members found challenging, resulting in a suboptimal response rate (54%). Many individuals from lower socio-economic backgrounds rely on Smartphones to access the internet [45] and other researchers have documented challenges associated with using online platforms to engage such community members during the COVID-19 pandemic [46]. Nonetheless, online platforms open new opportunities for reaching community members given they can assist in removing barriers related to transportation and time required to travel to locations where programs are offered. In future research aimed to examine the effectiveness of this program, when both in-person and virtual program delivery are potential options, it may be helpful to consider the respective benefits and drawbacks of each approach in order to best meet community members’ needs.

Even with a strong relationship between key program partners, we encountered challenges with regard to outreach in the community when it came to identifying venues for program delivery. The promotor/as with whom we worked have a long history of delivering programs in schools and churches. However, during the COVID-19 pandemic, there were considerable barriers to accessing these settings. This proved to be a significant logistical challenge, but also an important learning opportunity for our team. In future endeavors, we will work to obtain buy-in from a range of community agencies early on in our efforts to ensure their involvement and participation, which we recognize as vital to reaching the audiences for whom our programs are intended. We will also explore opportunities to engage with service professionals who are directly employed by schools, such as school counselors or teachers, to ensure that program delivery can be sustained even when unforeseen barriers to bringing outside services into the school environment arise. It may also be feasible to engage other school staff, such as administrative professionals or teaching aids, in the role of promotor/a. Individuals in these roles often live in the local community and share the language and background of community members, making them a good fit for the promotor/a role.

Our newly developed materials proved to be effective in preparing promotor/as for program delivery and in bringing valuable health information to the community. It will be important for us to consider the value of splitting lengthy training sessions into multiple days in the future. While this format provided the opportunity to complete and discuss homework following the first training session, it did result in some drop off such that interested promotor/as were unable to complete the full training. Feedback obtained indicates that materials were deemed appropriate and accessible by promotor/as and community members alike. Among promotor/as, results demonstrate increased knowledge regarding vaping and its adverse health effects as well as increased confidence in their ability to educate community members about vaping. Community members who participated in promotor/a-led education sessions demonstrated a strong understanding of the dangers posed by vaping as well as an intention to discuss the issue with their children. Prior literature has described the feasibility and effectiveness of promotor/a-led programming in the area of tobacco cessation [47–50]. Although the prelimary results presented here are promising, resources and time devoted to more rigorous program evaluation are needed to examine the effectiveness of promotor/as in the area of tobacco prevention and control, including with regard to vaping. Additionally, more rigorous future research specific to this program will be needed adequately consider potential variation in program effectiveness depending on community members’ demographic characteristics, including the particular region of the County in which they reside. Other limitations of this preliminary work that we hope to address in future research include the need for more rigorous evaluation with parents who participate in the program, including pre- and post-program surveys that assess changes in knowledge as well as longer-term follow-up to examine whether parents act on their intentions to discuss vaping with the children.

We are pleased to share that use of our materials and approaches currently continues among the promotor/as who were involved in the project. Without ongoing financial support however, the promotor/as whom we trained are unlikely to have the bandwidth to prioritize this program into the future. Issues related to securing ongoing funding for promotor/a programming have been discussed elsewhere in the literature [51]. With regard to the current program, we will explore opportunities to train and engage promotor/as in paid roles through partner institutions such as schools and social service agencies as one mechanism to ensure program sustainability. The high level of interest in this project conveyed by these program participants and by other promotor/a groups across the state and nation suggests that, with appropriate funding, widespread dissemination and use of this promising and much needed program would be possible. Given the importance of promotor/a-led programming for cancer prevention and control among Latinx populations, the largest ethnic minority group in the U.S., materials such as these that can be used for program delivery in the community are critically important. Additionally, whereas the preparation of those responsible for program delivery and dissemination is often noted in the description of community health interventions, specific details regarding materials utilized and steps taken to prepare these change agents in order to maximize the effectiveness of their efforts are less frequently provided. Simlarly, assessment of these steps is frequently lacking. We are hopeful that the detailed information provided about these processes as they related to this project will be informative to others wishing to engage in similar work. Establishment of mechanisms for cancer centers to share resources developed as well as details related to the development process could significantly enhance cancer prevention and control program delivery for diverse populations at the national level.

### Supplementary Information

Below is the link to the electronic supplementary material.

Supplementary file1 (PDF 1859 kb)

Supplementary file2 (PDF 17478 kb)

## Data Availability

The data generated and analysed during the current project are not publicly available due to concerns related to the protection of participant privacy. All reasonable data requests will be considered by the corresponding author on a case by case basis.
