# Supplementary material for: Adapting E-cigarette prevention programming to reach the latinx community
Source: Cancer Causes Control. 2023 Oct 9;35(3):405–16. doi: 10.1007/s10552-023-01796-7 (PMC10838817; doi:10.1007/s10552-023-01796-7)
Supplement: Supplementary file 1 — Supplementary file1 (PDF 1859 kb) [file 10552_2023_1796_MOESM1_ESM.pdf]

UNITED IN THE PREVENTION OF ELECTRONIC CIGARETTES AND VAPING

Training Manual

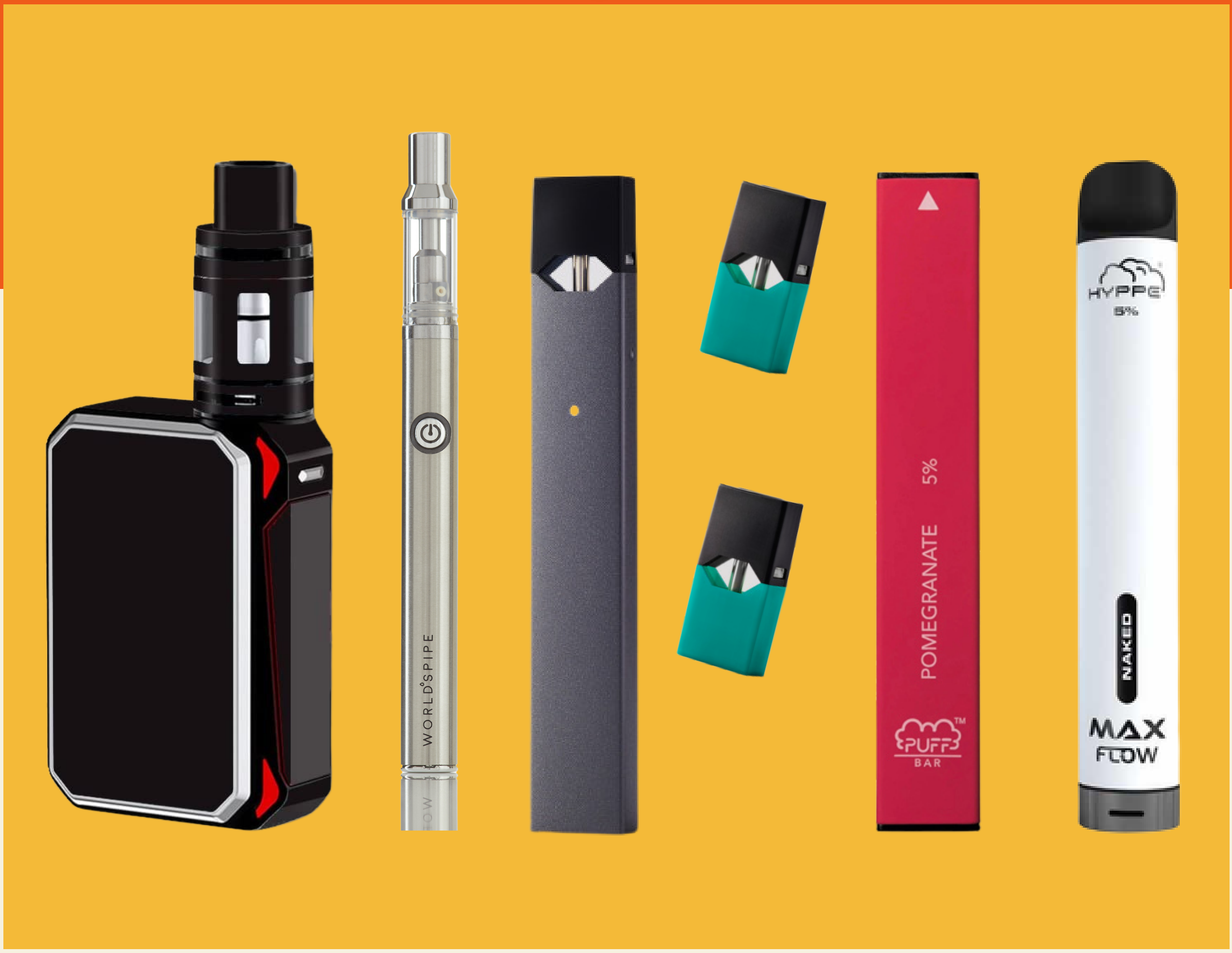

UCLA Jonsson Comprehensive Cancer Center

Visión y Compromiso™

UCLA KAISER PERMANENTE®
CENTER FOR HEALTH EQUITY

Module 1	*Additional notes:*

Electronic cigarettes

What are e-cigarettes?

- Battery-powered devices that heat a liquid and produce an aerosol that is inhaled into the lungs.

There are many types of e-cigarettes. They are always changing.

What is in e-cigarettes?

- Ingredients may contain:
  - **Flavors** (artificial flavors)
  - **Nicotine** (extracted from the tobacco plant)
  - **Cannabis** (marijuana)
    - THC - psychoactive chemical in marijuana
  - **Aerosol and toxic chemicals**

Flavors (artificial flavors)

- More than 15,000 flavors
  - Hide the taste of nicotine
  - Decrease the smell of cannabis

Nicotine (extracted from the tobacco plant)

Cannabis (marijuana)

Aerosol and toxic chemicals

- The smoke created by e-cigarettes is not "water vapor" that evaporates.
- It is an aerosol that can leave a residue of toxic substances inside the body.

The use of e-cigarettes is called:

"vaping" and some young people even refer to it as "smoking".

E-cigarettes have many names:

- "Pens"
- "Vapes"
- "e-cigs"

The most popular e-cigarettes among teens:

- Pod systems - have two parts: a battery and a pod with an e-liquid (pre-filled or refillable)
  - Ex: JUUL - has been very popular
- Single-use disposables
  - Ex: Puff Bar - a popular brand
- Vape Pens - pen-like: use a battery and can be charged
  - They are filled with an e-liquid

Where do young people get e-cigarettes?

Ways young people get e-cigarettes:

- Other people (friends, family, or people they know)
- Websites and online orders
- Social media
- Vape or tobacco stores, gas stations, convenience stores, or liquor stores
- Illicit market

Module 2

In the United States:

- E-cigarettes are hooking a new generation of tobacco users, threatening efforts to stop cigarette use.
- Among all youth:
  - E-cigarettes are the most commonly used tobacco product.

Vaping Among Latino Youth in 2020

- Among all middle school students, Latino youth have the highest rate of e-cigarette use.
- Latino youth have the highest rate of cannabis vaping.

Reasons Why Youth Use E-cigarettes

Top reasons among Latino youth:

- Curiosity
- Flavors
- Influence of friends or family

Additional reasons among all youth:

- Relieve stress
- Peer pressure
- Are hooked or have an addiction
- Accessibility and marketing

Most Popular E-cigarette Flavors Among Youth

- The most common flavors:
  - Fruit
  - Mint
  - Menthol
  - Sweets and Desserts

Additional Notes:

Scan for more information and resources

or visit: tinyurl.com/e-cigsresources
