# Supplementary material for: Adapting E-cigarette prevention programming to reach the latinx community
Source: Cancer Causes Control. 2023 Oct 9;35(3):405–16. doi: 10.1007/s10552-023-01796-7 (PMC10838817; doi:10.1007/s10552-023-01796-7)
Supplement: Supplementary file 2 — Supplementary file2 (PDF 17478 kb) [file 10552_2023_1796_MOESM2_ESM.pdf]

SAMPLE OF FLIPCHART CONTENT

United in the Prevention of Electronic Cigarettes and Vaping

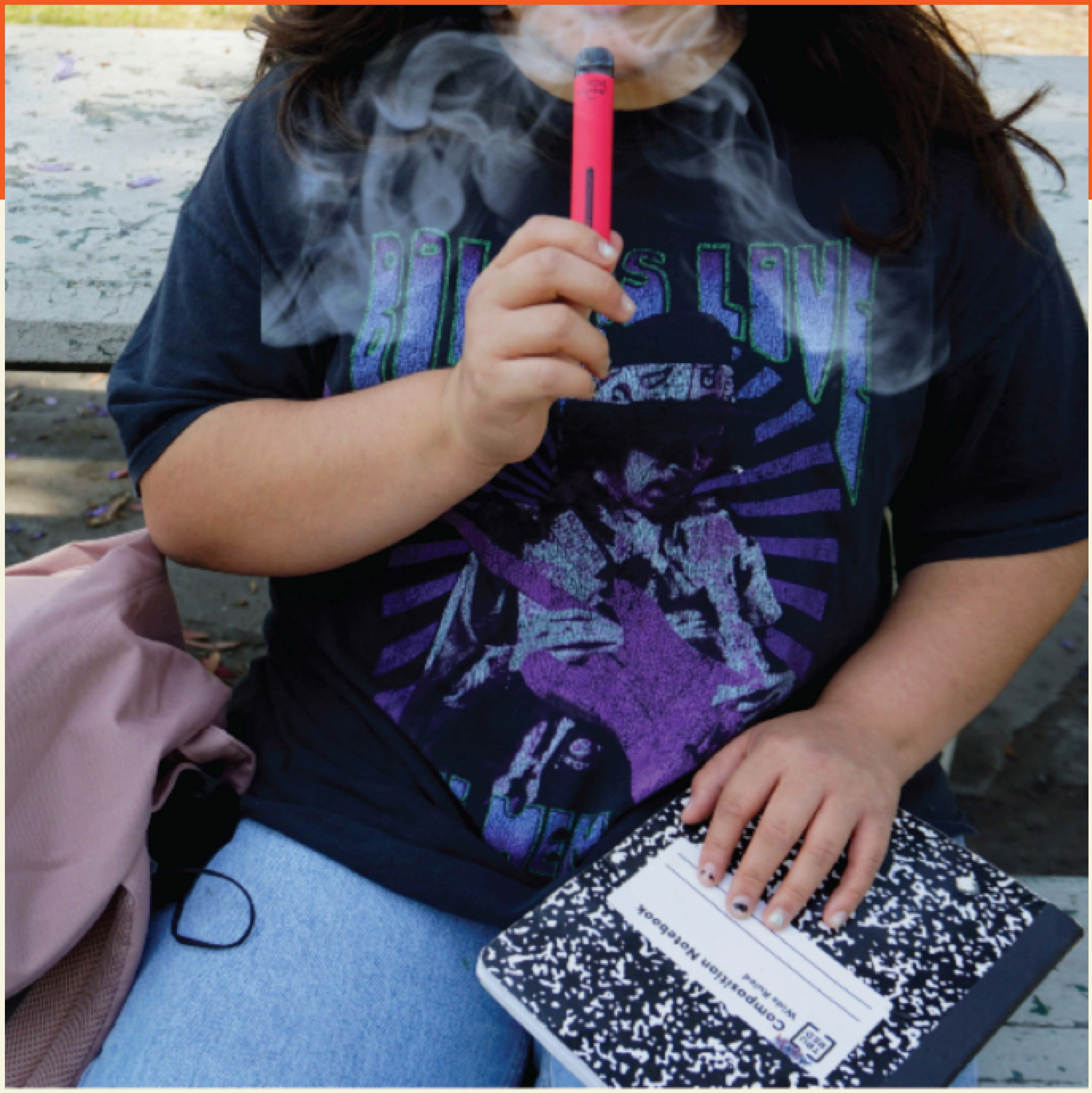

Visión y Compromiso™

Visión y Compromiso

UCLA Jonsson Comprehensive Cancer Center

UCLA KAISER PERMANENTE®
CENTER FOR HEALTH EQUITY

Questions to start the talk:

- What have you heard about e-cigarettes and vaping?
- What would you like to learn about e-cigarettes and vaping?
- Have you seen it in your community?

What are electronic cigarettes?

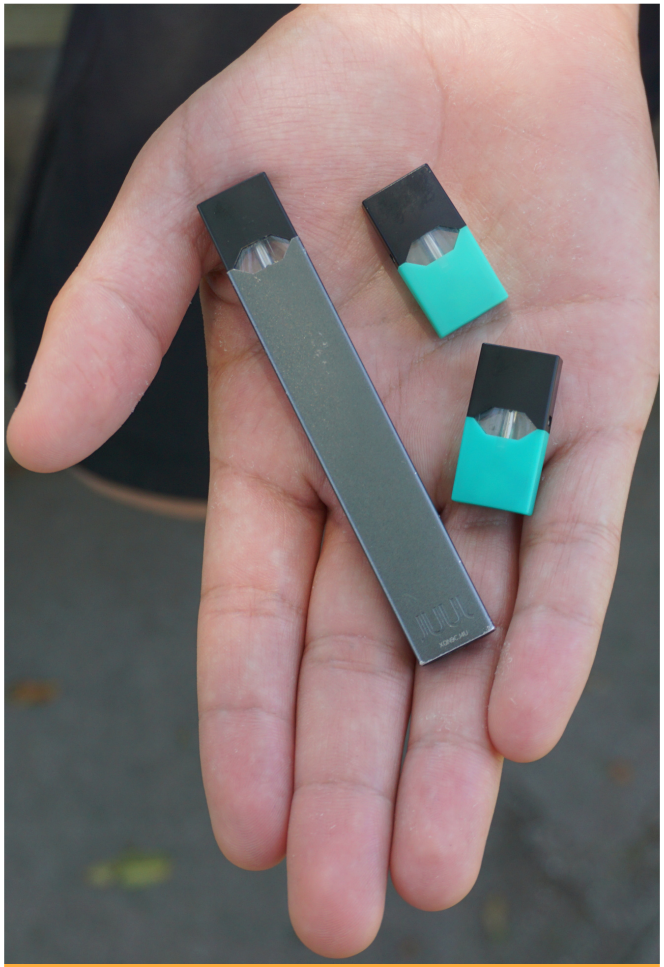

Pod systems

JUUL

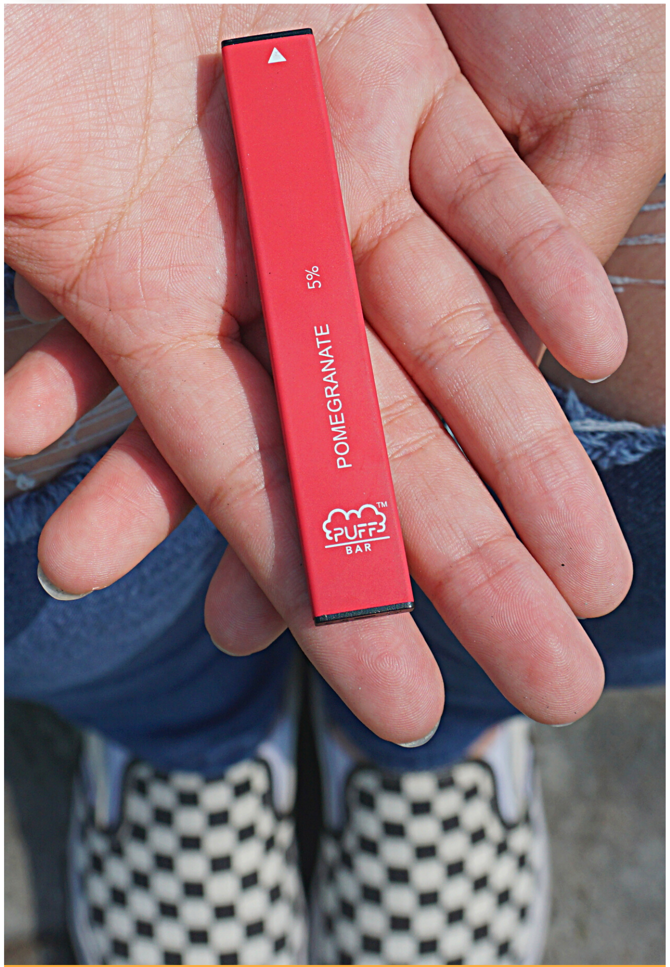

Disposables

Puff Bar

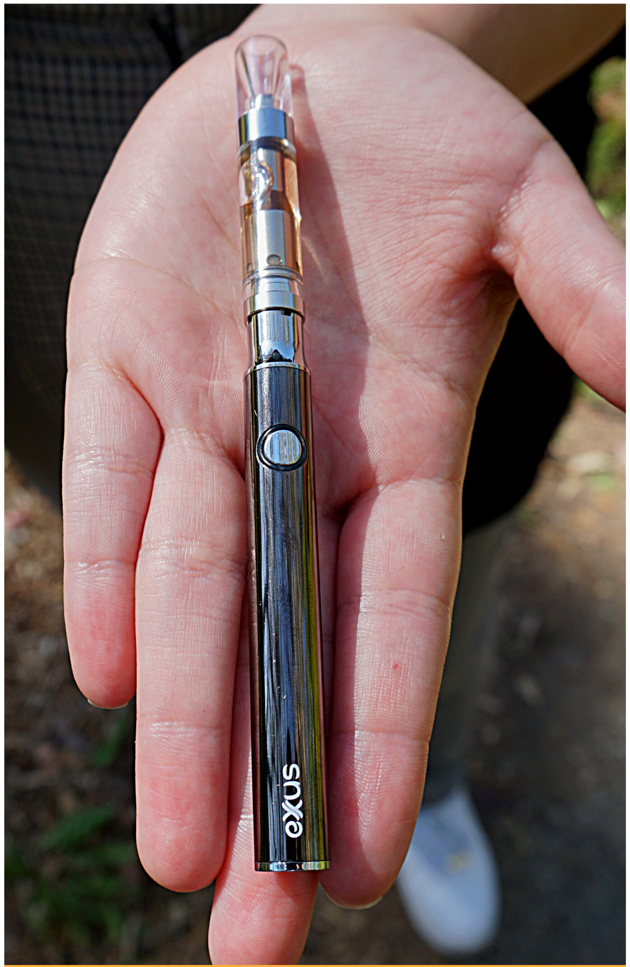

Vape pens

What are electronic cigarettes?

- Battery-powered devices that heat a liquid and produce an aerosol that is inhaled into the lungs.

There are many types of e-cigarettes. They are always changing.

The most popular e-cigarettes among teens:

- Pod systems - have two parts: a battery and a pod with an e-liquid (pre-filled or refillable)
    - Example JUUL - has been very popular
- Single-use disposables
    - Example Puff Bar - a popular brand
- Vape Pens - pen-like: use a battery and can be charged
    - They are filled with an e-liquid

Other names:

- "Vapes"
- "Pens"
- "e-cigs"

What is in e-cigarettes?

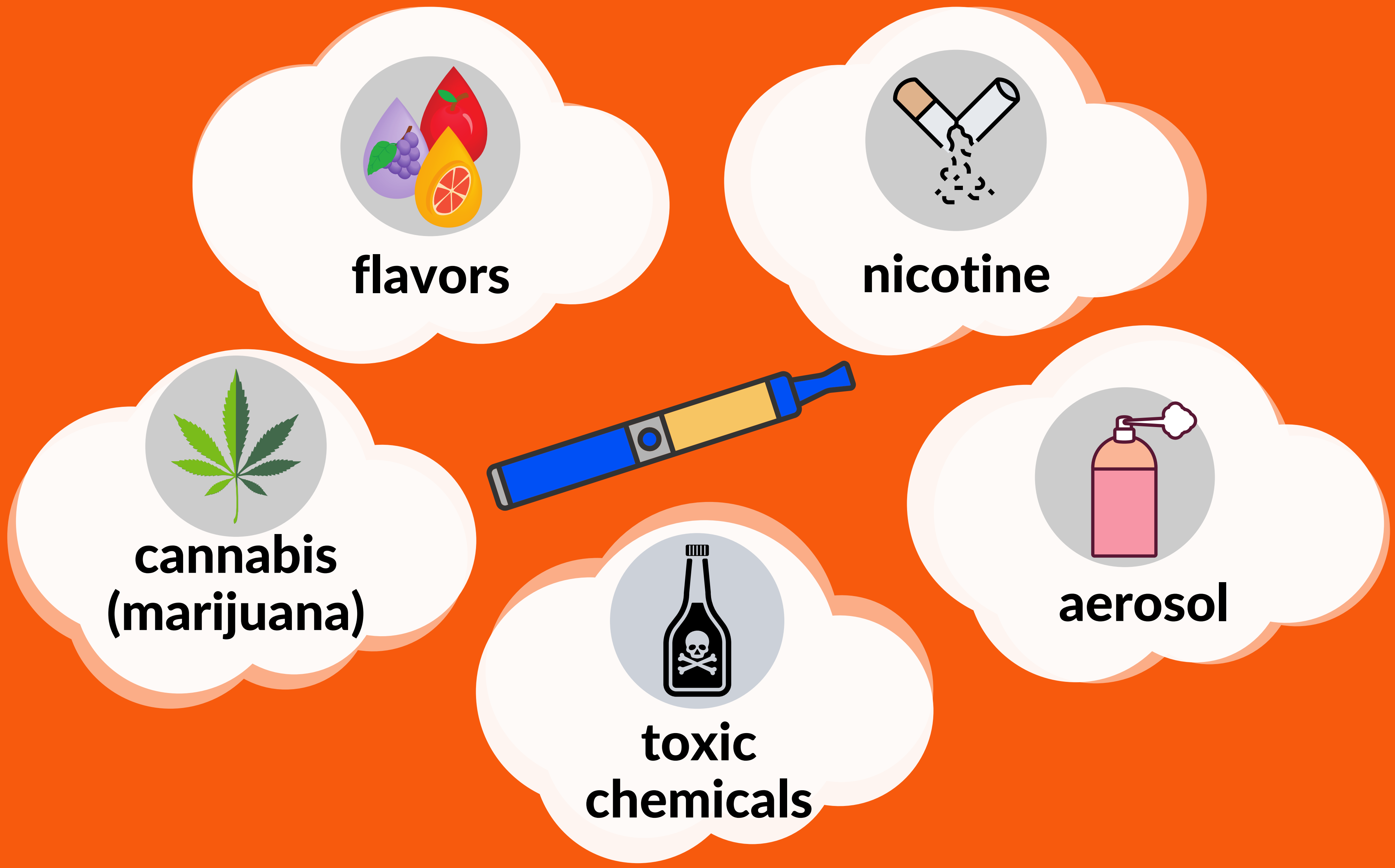

What is in e-cigarettes?

- Ingredients may contain:
  - Flavors (artificial flavors)
  - Nicotine (extracted from the tobacco plant)
  - Cannabis (marijuana)
    - THC - psychoactive chemical in marijuana
- Toxic chemicals -
  - Many of the same chemicals found in traditional cigarettes.
  - Chemicals related to lung disease, cancer, and reproductive health problems.
    - Some of them are found in other products like nail polish remover or pesticides.
- The smoke created by e-cigarettes is not "water vapor" that evaporates, it is an aerosol that can leave a residue of toxic substances inside the body.

How can vaping affect my child?

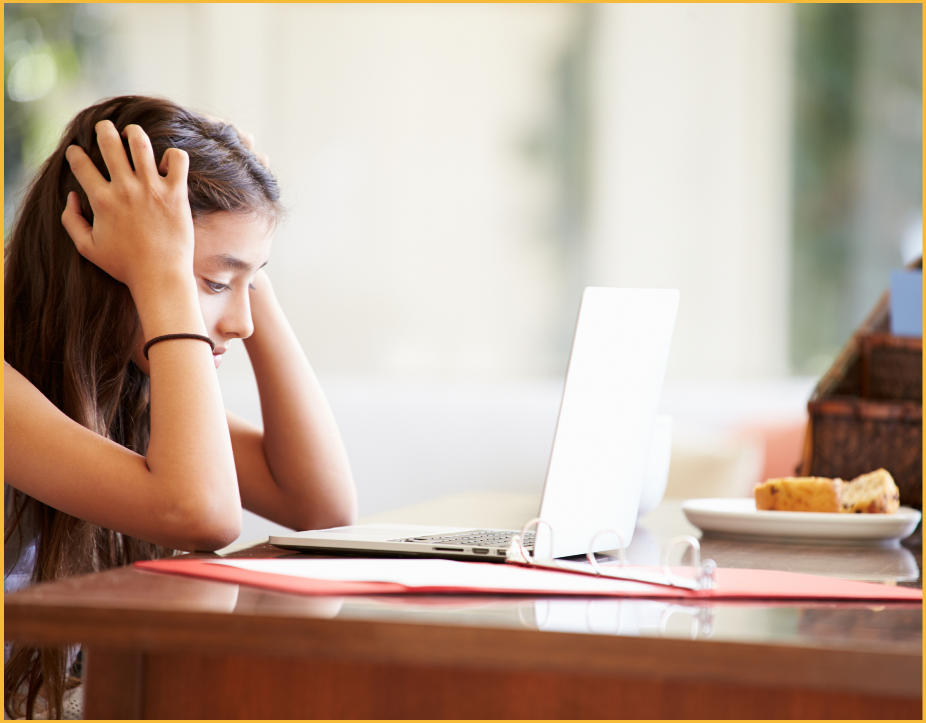

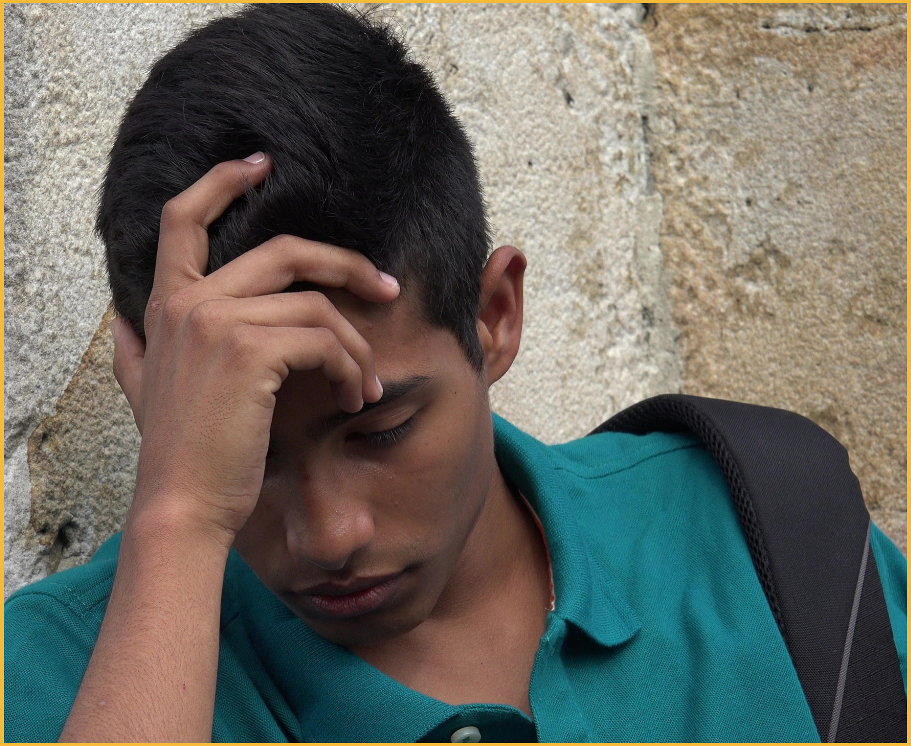

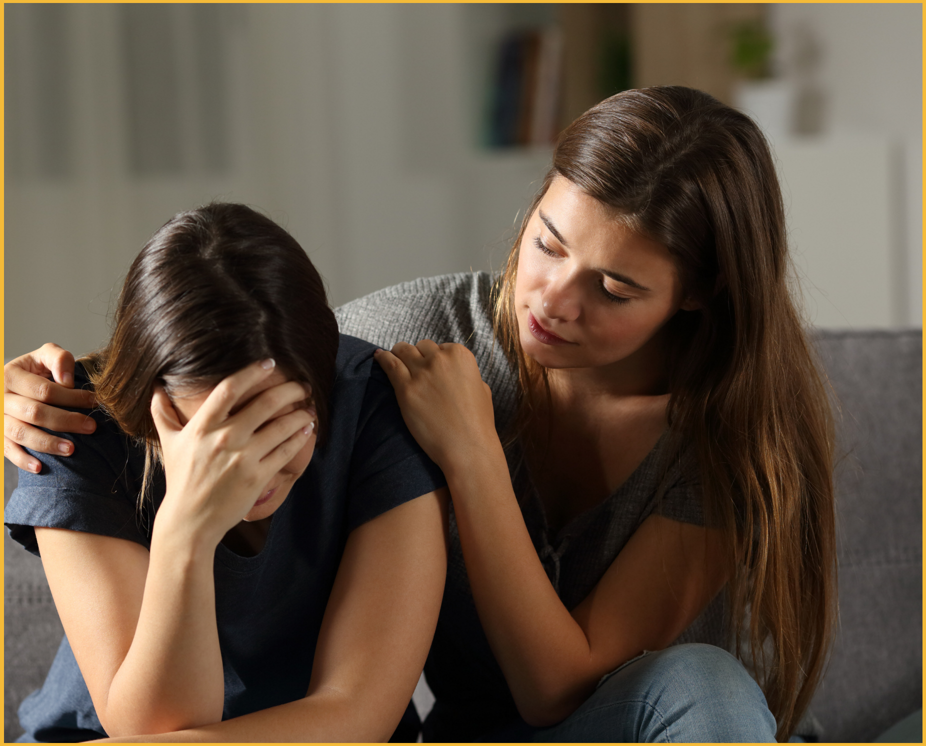

There is an increased risk of addiction:

- The likelihood of addiction increases for those who start young

Other risks include:

- Anxiety
- Mood swings
- Irritability
- Impulsiveness

The use of e-cigarettes can lead young people to:

- Smoke traditional cigarettes
- Use marijuana

Recognize the signs

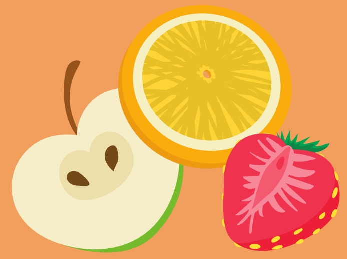

Fruit or sweet smells

Behavior changes

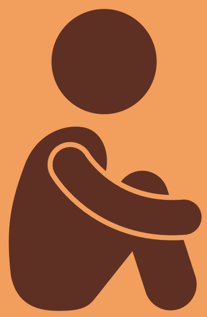

Unknown items or products

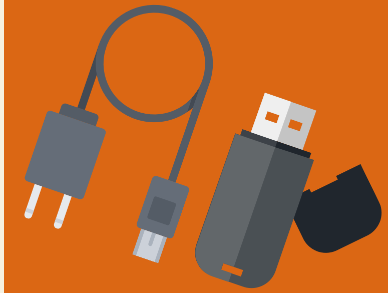

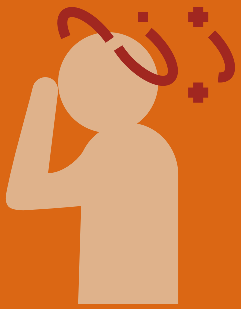

Physical symptoms

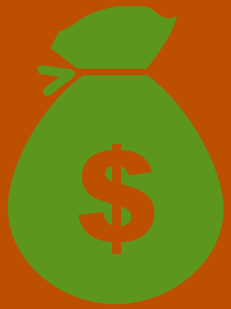

Spending more money than usual

Mood swings

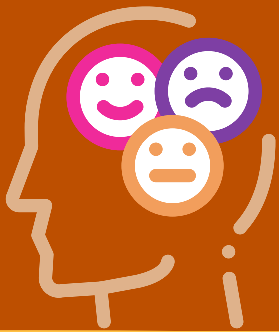

Recognize the signs:

- Fruit or sweet smells
- Unknown items or products
- Spending more money than usual or making unexplained purchases
- Changes in behavior:
    - Reserved attitude
    - Closed door, isolating themselves
    - Frequent excuses to go to the restroom, or go outside
- Physical symptoms:
    - Cough
    - Headaches and nausea
- Increase in mood swings, irritability, anxiety, impulsivity, or learning difficulties.

What can parents do?

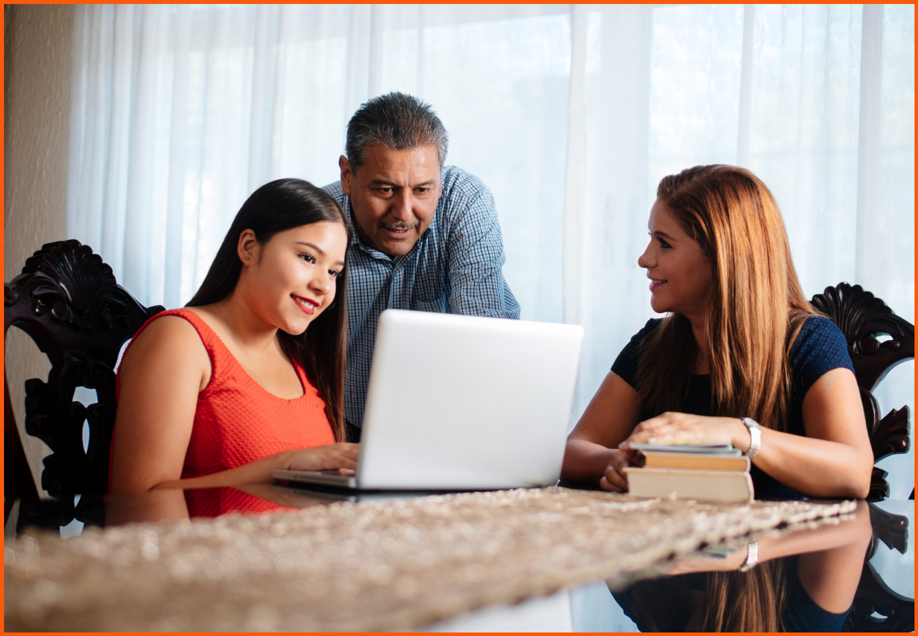

Have conversations often

Be ready to listen, not lecture

Ask for the help of a trusted adult

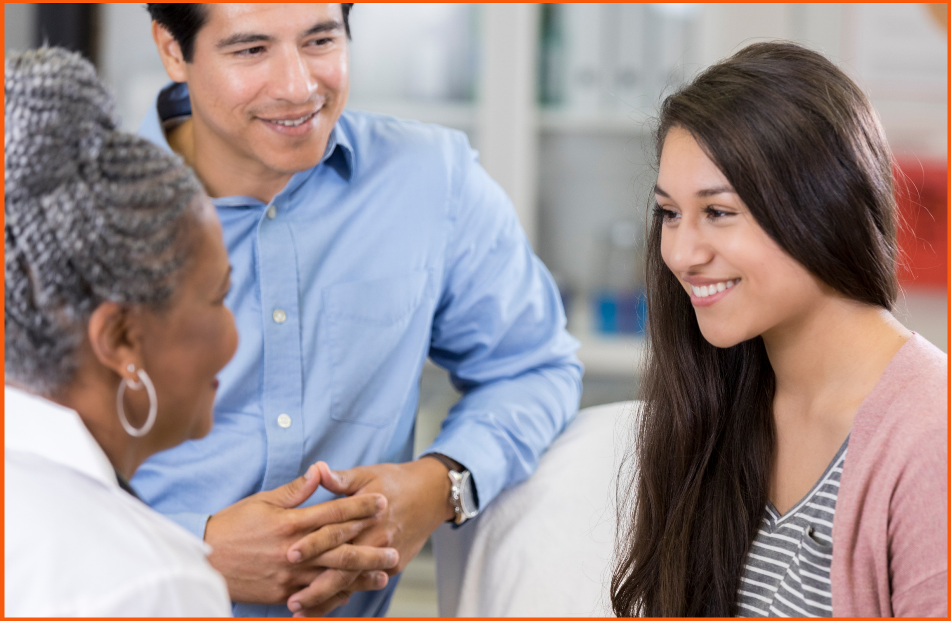

Actions that parents or primary caregivers can take:

- Model healthy behaviors

- Have conversations often about this topic
  - Find good opportunities to talk
  - Be ready to listen, not lecture

- Ask for the help of a trusted adult or suggest that your child speak with a trusted adult
  - Family member
  - Doctor
  - Teacher
  - Coach
  - Promotor
